# From Tradition to Technology: Robotic Artificial Intelligence in Dental Implantology

**DOI:** 10.7759/cureus.73340

**Published:** 2024-11-09

**Authors:** Kaitlyn P Huynh, Gibran Mangui

**Affiliations:** 1 Polygence, Notre Dame High School, San Jose, USA; 2 Dentistry, University of Maryland, Baltimore, USA

**Keywords:** accuracy of implant placement, dental implants, dynamic navigation system, robotic-assisted surgery, robotic dental implant surgery

## Abstract

Dynamic navigation and haptic robotic systems are revolutionizing dentistry by significantly improving implant placement precision, especially in the maxillofacial region where safeguarding nerves, vessels, and vital structures is crucial. This article examines the impact of X-Guide dynamic navigation and Yomi robotics on implant surgery outcomes, comparing these technologies to traditional freehand methods. The X-Guide dynamic navigation system and Yomi robotics have both been proven to enhance accuracy, with both achieving mean deviations of around 1 mm from pre-planned positions. However, the high costs associated with these technologies may limit access for many practitioners. Despite these challenges, both systems facilitate the completion of complex cases with minimal deviations. In addition, their applications are expanding beyond implantology into areas like endodontics, further demonstrating their potential in modern dental practices.

## Introduction and background

Dental implants are frequently utilized in oral rehabilitation because they offer highly successful, long-term solutions that maintain bone structure and provide support. They are typically made of titanium and are surgically embedded into the jawbone beneath the gum tissue to help address both partial and complete tooth loss [1]. 

Brief history of dental implants

The earliest documented use of dental implants dates back to 600 AD when the Mayan civilization innovatively employed shell materials to replace missing mandibular teeth [1]. Radiographs of Mayan mandibles reveal dense peri-implant bone formation similar to bone morphology around modern implants. In the 1940s, Formiggini, known as the Father of Modern Implantology, and Zepponi introduced an endosseous implant with a spiral stainless steel design [1]. Later, Dr. Perron Andres enhanced the design by adding a solid shaft. As implants continued developing in the 1940s, Swedish researcher Dahl pioneered subperiosteal (on the bone) implants. In 1967, Dr. Leonard Linkow introduced blade implants, including the Ventplant implant, enabling placement in both the maxilla and mandible [1,2]. 

Brånemark, in 1952, discovered that titanium could bond to bone when titanium chambers were placed in rabbit femurs for blood flow. He found that they became firmly affixed and could not be removed. Branemark coined osseointegration as “a direct structural and functional connection between ordered, living bone, and the surface of a load-carrying implant” [1,2]. Initially tested in 1965, Brånemark’s two-stage threaded titanium root-form implants, termed fixtures, were first applied in a patient with severe jaw deformities and dental anomalies. Four implants were placed in the mandible, integrating within six months and maintaining functionality for 40 years [2]. 

Implant success

In dentistry today, dental implants have been shown to have a 10-year success rate of 90 to 95%. Although widely favored by dentists, complications with dental implant placement and care continue to pose clinical challenges [3]. Periodontal diseases, often stemming from inadequate brushing and flossing, can contribute to the development of peri-implantitis by promoting plaque accumulation and hardening of biofilms. Peri-implantitis is an inflammatory condition induced by bacterial biofilms, affecting both the soft and hard tissues around dental implants. It resembles periodontitis but occurs around implants rather than natural teeth. Symptoms of peri-implantitis typically include bleeding on probing, suppuration, and progressive bone loss beyond normal physiological remodeling. In severe cases, this condition can lead to implant failure and may require its removal [4].

In particular, multiple studies indicate that smoking elevates implant failure compared to nonsmokers, with reported failure rates ranging from 6.5% to 20%. While nicotine-induced vasoconstriction can cause bone loss, other studies show that implant failure stems from peri-implant tissue exposure to tobacco smoke rather than issues with healing or osseointegration [5]. 

Patient non-compliance is another challenge, as implants are vulnerable to plaque-related diseases. Adequate plaque control and regular dental follow-ups are essential to prevent peri-implantitis. Age-related factors, such as reduced manual dexterity and visual acuity, also affect implant outcomes [6].

Important anatomical features

Due to the distinct nature of dental implant surgery compared to other dental treatments, such as restorative and minimally invasive procedures, a comprehensive knowledge of the anatomy in the maxillofacial region, including the maxilla and mandible, is crucial. 

**Figure 1:**
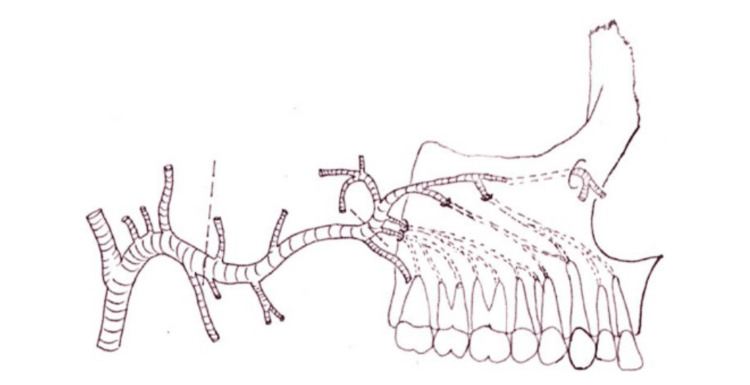
The posterior superior alveolar artery enters the alveolar foramen and runs through the alveolar canals. Reproduced without modification from reference [7] under the Creative Commons License (CC BY-NC-ND 4.0). For further details, please visit the Creative Commons License (https://creativecommons.org/licenses/by-nc-nd/4.0/).

The posterior superior alveolar artery, which originates from the maxillary artery, enters the alveolar foramen before it travels through the alveolar canals to supply the maxillary molars, gingiva, and buccal mucosa (Figure 1). These canals, visible in CT scans, require careful consideration during pterygoid implant placement or tissue harvesting to avoid damaging the artery. This artery also supplies the lateral wall of the maxillary sinus and requires particular caution during sinus lift procedures, a technique used to provide sufficient bone in the maxillary (upper jaw) sinus to allow for the placement of dental implants [7].

**Figure 2:**
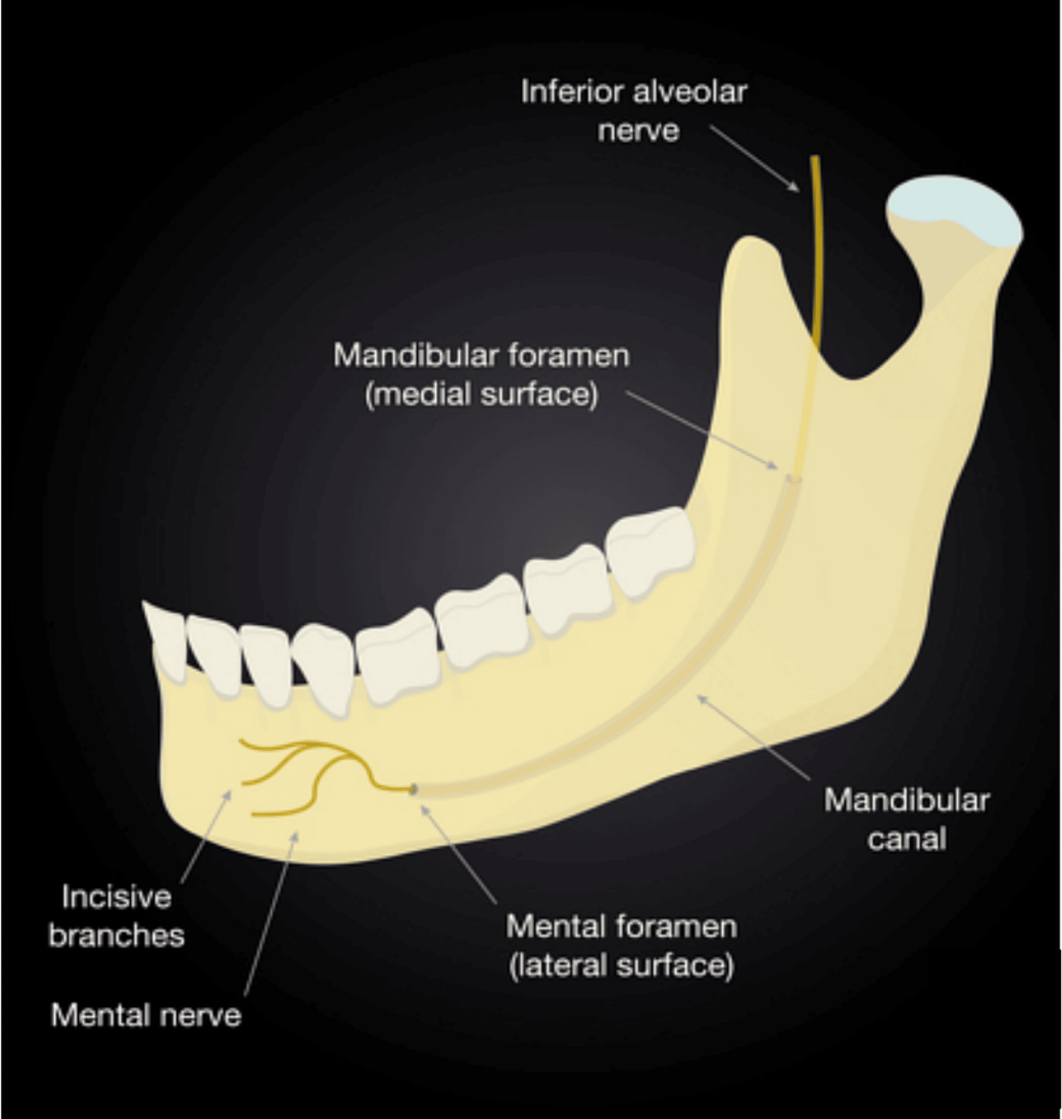
The inferior alveolar nerve entering the mandible through the mandibular foramen and eventually exiting via the mental foramen. Reproduced without modification from reference [8] under the Creative Commons License (CC BY-NC 4.0). For further details, please visit the Creative Commons License (https://creativecommons.org/licenses/by-nc/4.0/).

The mandibular canal (Figure 2), situated within the mandible, houses the inferior alveolar nerve (IAN) and artery. It begins at the mandibular foramen and extends to the mental foramen in the front. The IAN splits into the incisive and mental nerves. Maintaining the integrity of the IAN is critical as injuries to the nerve during implant procedures can lead to severe complications: for example, it can lead to altered sensation, paresthesia (tingling or numbness), or even complete nerve function loss in the chin, lower lip, and the skin overlying the anterior mandible [8]. 

The mental foramen is another crucial anatomical point, as the mental nerve is susceptible to damage during various dental procedures, including endodontics, extractions, and implant placement in the lower premolar and anterior mandible region. In addition, the anterior loop of the mental nerve, an anatomical variant, has been studied along with its distance from the mental foramen. Such injuries to the mental foramen can result in sensory disturbances in the lower third of the face [9].

**Figure 3:**
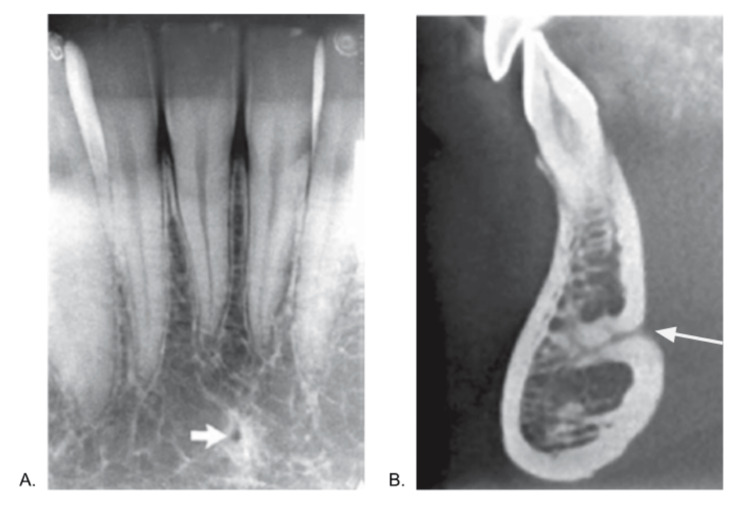
a: The lingual foramen, indicated by the arrow, is the small rounded opening shown in the intraoral periapical view. b: In the cross-sectional cone beam computed tomography image, the lingual foramen extends deep into the mandible from the lingual surface. Reproduced from reference [10] under the Creative Commons License (CC BY-NC-SA 4.0). Labels (A and B) were adjusted from the original labels to align with the current study, and an arrow was added to panel B. No other modifications were made to the image. For further details, please visit the Creative Commons License (https://creativecommons.org/licenses/by-nc-sa/4.0/).

​​The lingual foramen (Figures 3A, 3B) is a key structure in the anterior mandible and is typically found along the midline, either above or below the genial tubercle [10]. Its location near the sublingual space is associated with a significant bleeding risk during surgery, making damage to the lingual foramen potentially life-threatening [11].

Implant planning

Obtaining an accurate imprint of the dental arch or area is critical to ensure surgical success and proper placement of a prosthesis. Implant planning begins with capturing precise 3D images of the jawbone obtained from computed tomography (CT) or cone beam computed tomography (CBCT) scans. CBCT is a radiographic imaging method that provides three-dimensional (3D) imaging of hard tissue structures, such as the jaw and teeth. CBCT has significantly lower radiation exposure than conventional CT scans (68 µSv versus 600 µSv). CBCT employs an X-ray beam that scans 360 degrees around the patient, capturing single projection images, or “basis” images, at specific intervals. Advanced software programs are applied to the projection data to create a comprehensive 3D volumetric dataset in the axial, sagittal, and coronal planes [12].

After this step, an intra-oral scan of the patient is performed. Intraoral scanning is based on 3D systems that capture detailed information on the shape and size of dental arches and the patient’s teeth and soft tissues. Intraoral scanning is recognized as a superior alternative to conventional impressions as it reduces the risk of distortion associated with traditional impression materials, allows for quick error rescanning, and alleviates the discomfort and time-consuming nature of patients [13,14]. The 3D data from the CBCT scan and intraoral scan are aligned to produce a complete digital model of the patient’s oral anatomy. Combining the bone structure details from the CBCT scan with the dental arch specifics from the intraoral scan, this comprehensive model facilitates two primary guided surgery systems: static guidance and dynamic navigation [15].

"Static" refers to a system where the implant position is fixed according to the predetermined implant placement. It does not allow for real-time adjustments during the procedure. A static system employs computer-aided design/computer-aided manufacturing (CAD/CAM) stents with metal tubes and a surgical system. Specifically, the 3D data from the CBCT and intraoral scans are imported into CAD software for detailed planning. CAM technologies then use this information to create a precise surgical guide through rapid prototyping methods such as 3D printing and stereolithography [16]. These guides fit securely onto the existing dentition or edentulous area during surgery and help direct the surgeon to the predetermined sites to ensure the accuracy and protection of vital structures. However, drawbacks of this system include instrument misalignment due to restricted mouth opening, potential guide fractures, and the inability to make intraoperative adjustments during placement [15,17]. 

On the other hand, dynamic navigation is a computer-guided navigation system that helps the clinician in real-time during the implant positioning through visual imaging tools on a monitor [15]. X-Guide (X-Nav Technologies, Pennsylvania) is a leading example of dynamic 3D navigation systems. These systems utilize optical tracking with passive or active arrays to track the position of the dental drill. Passive systems reflect light to cameras, while active systems emit light tracked by cameras. Some advantages to dynamic navigation over static systems include real-time adjustments, continuous visualization of the drill in three dimensions, and avoiding issues like guide displacement. However, some limitations include sensitivity to reflections, the need for a clear line of sight between the tracking device and cameras, higher cost, and a significant learning curve for uses [16].

Brief introduction to robotic-assisted implant surgery (RAIS)

RAIS enhances flexibility, stability, and accuracy in placing implants, addressing challenges such as challenging working positions to obtain direct vision, operator fatigue, and human errors associated with traditional methods. Although robotic systems have a longer history in medicine, they have also rapidly gained traction in dental surgery, leading to their integration across multiple dental disciplines, including prosthodontics, oral surgery, and implantology. As the demand for greater precision in dental implants grows, these robotic systems are being recognized for their advanced capabilities, such as robotic intelligence, machine vision, multi-sensor integration, and 3D visualization [18].

Dental robotic systems utilize haptic feedback, which refers to touch-based sensations that provide real-time information about interactions with the surgical site, along with robotic guidance to ensure precise implant placement. These systems offer real-time visualization and tactile feedback, providing the static benefit of physical constraints and the dynamic advantages of same-day surgery and intraoperative adaptability [19].

Dental implant robotics have evolved to include various systems with differing levels of automation and user interaction. A typical dental implant robot comprises three primary components: a robotic arm, a visual system, and a central control system. As outlined by Troccaz et al., these systems are categorized based on the level of user interaction during tool motion. Active robots autonomously carry out pre-planned motion, while passive robots operate manually, semi-active robots allow for constrained motion, and teleoperated robots hold the tool but are remotely controlled by the user [20]. Yomi (Neocis, Inc., Florida) is the first haptic robotic-guided system to receive FDA clearance, designed to drill using a coordinate system mapped onto teeth autonomously. Other dental implant robot systems have been introduced following Yomi’s introduction including the Remebot and Dentbot [18].

This paper will systematically review and analyze advancements in robotic-assisted dental implant surgery, focusing on Yomi and X-Guide. By examining the applications, precision, and efficiency of these technologies, this review aims to offer insights into their potential to shape the future of implant dentistry.

Materials and methods

This systematic review focused on the following question: In patients requiring implants, how has robotic artificial intelligence affected implant surgery outcomes and accuracy in the past and present?

The definitions of population, intervention, comparison, and outcome (PICOs) were developed based on the focused question. They were defined as follows: in patients requiring dental implants (population), how has robotic artificial intelligence (intervention) in the past and present (comparison) affected implant surgery (outcomes).

An electronic search was conducted without time or language restrictions using PubMed, Google Scholar, ResearchGate, and other article databases. The reference lists of included studies and relevant reviews were also searched for other potential studies. The following keywords were used in our search: Robotic Dentistry, Dental Implants, Cone-Beam Computed Tomography, Navigated Surgery, Dynamic Navigation, Haptic Robot Guided Systems, Robotic-Assisted Implant Surgery, X-Guide, Yomi.

## Review

Freehand method

Due to limited technology, dental implants have historically been placed freehand. This is called the freehand method because the surgeon relies solely on radiographs to evaluate the optimal location, depth, and angle for inserting implants without robotic assistance or static guides. Accuracy refers to the positional or angular deviation between the actual and planned implant positions [21]. Thus, understanding the outcomes and complications associated with different methods is crucial. 

Arisan et al. found that 54.65% of implants using the freehand method were classified as inadequately positioned, whereas this rate decreased to 15.78% with a mucosa-supported drilling template [22]. Other studies have similarly documented deviations between the virtual and actual implant positions using the freehand method: in a cadaver study, average deviations were 1.43 mm (range: 0.65-2.31 mm) at the implant shoulder, and 2.20 mm at the apex, with angular deviations ranging from 3.08° to 14.98° [23]. Likewise, Varga et al. observed average deviations of 1.82 mm (range: 0.56-5.38) at the implant shoulder and 2.43 mm (range: 0.54-4.83 mm) at the apex, with angular deviations ranging from 0.71° to 21.30° [24]. Aydemir and Arisan observed an average angular deviation of 10.04° (range: 2.19°-20.42°) using the freehand method, underscoring the consistent findings of significant angular deviations in clinical studies [25]. 

The precision attainable through freehand implant placement can vary significantly based on the surgeon's proficiency. In an in vitro study by Jobi-Garcia et al., two researchers (one novice, one experienced) placed 36 implants in six resin mandible models using dynamic navigation and the freehand method. Experienced implant surgeons showed an average angular deviation of 6.69°, compared to 12.66° for less experienced surgeons, when comparing the actual implant position on postoperative CBCT to the virtual preoperative placement [26]. However, angled abutments can assist in correcting implant angulations when placing a prosthesis. Depending on the implant company, angles from 17 to 35 degrees can be corrected with angled custom abutments. 

Pioneering surgical robotics

The development of dental implant robots has progressed significantly over the past two decades. In 2002, Boesecke et al. introduced the concept of an implant surgery robot at the University Hospital of Heidelberg: a prototype robot system featuring a 700 mm reach and operated via PC-based TomoRob software (Medical Intelligence, Germany). The system's function included maintaining the drill template alignment by preoperative plans [27]. In 2017, an autonomous dental implant robot was developed to tackle the significant shortage of skilled dentists in China (Collaboration between the Fourth Military Medical University Hospital in Xi’an and the robot institute at Beihang University; China). It has shown excellent outcomes, with data showing an error of 0.2-0.3 mm [28].

Although advancements are ongoing in the development of autonomous robots for dentistry, robotic systems have been much more prevalent in medicine. The origin of robot-assisted surgery can be traced back to work at NASA in the mid-1980s when they developed a remotely controlled robotic system intended for surgical use in battlefield conditions and space missions [29]. In the year 2000, the FDA approved the first robotic system for laparoscopic surgery: the da Vinci Surgical System (Intuitive Surgical, California). The da Vinci robot is a remote extension of the surgeon's hands, utilizing robotic arms with surgical instruments to perform precise procedures through small incisions. Since its introduction in 2000, the da Vinci robotic system has evolved through several generations, incorporating advancements such as better anatomical exposure, advanced imaging systems, and tremor-reduction features [30]. 

X-Guide by X-Nav Technologies

**Figure 4:**
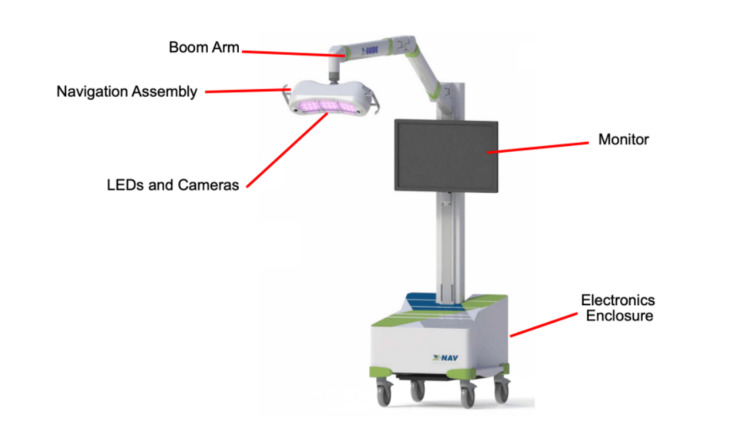
The X-Guide Surgical Navigation System’s setup. The mobile cart includes an electronics enclosure, LCD monitor, boom arm, navigation assembly, LEDs and cameras. Reproduced without modification from the FDA 510(k) Summary - K232148 (reference [32]). The FDA has waived all worldwide copyright rights under applicable law, allowing unrestricted use of the material.

The X-Guide Surgical Navigation System by X-Nav Technologies is a dynamic computer-assisted surgery system that relies on stereo triangulation from optical cameras, tracking the movement of two dynamic reference frames during surgery: one fixed to the patient's anatomy and the other to the surgeon's handpiece (Figure 4). The system processes this tracking data to deliver real-time guidance, a key benefit that enables surgeons to control drills with high precision [31].

Patient Tracker calibration determines the spatial relationship between the Patient Tracker and the patient's scan coordinates to ensure proper alignment. For partially edentulous patients, X-Clips with radiodense spheres are utilized, while edentulous patients use bone screws that serve as fiducials, effectively linking CT scans to their surgical anatomy. The X-Mark Registration process is suitable for both partially edentulous and edentulous patients. In this process, anatomical landmarks are marked directly on the CT scans by the surgeon, acting as fiducials. After identifying at least three landmarks, the surgeon uses a Probe Tool to link the Patient Tracker to the system, facilitating precise patient registration. The Digital Imaging and Communications in Medicine (DICOM) data is then used for implant planning, with X-Guide’s software offering features like parallel planning, virtual teeth, and nerve visualization. Calibration of the handpiece tracker and patient tracker to the 3D plan is done before surgery to provide live guidance during osteotomy. Postoperative CBCT scans are used to verify the accuracy of implant placement by comparing planned and final positions [32].

The X-Guide offers several advantages over traditional static guides. It facilitates same-day scanning, surgical planning, and surgery without requiring laboratory-fabricated stents, making the procedure more time- and cost-efficient. Additionally, the system allows surgeons to maintain an unobstructed view of the surgical site, as there is no stent blocking the field. Plans can be altered more easily during surgery, and the entire field can be visualized at all times. For challenging cases, such as those involving limited mouth opening, narrow interdental spaces, distal implant placements, or patients with a pronounced gag reflex, the X-Guide can be highly beneficial [31].

In a study by Emery et al. 2016, the X-Guide system demonstrated high accuracy on dental models. Eleven dentate maxilla and ten dentate mandible polyurethane models, each receiving a single implant to ensure independent measurements, were used to simulate surgical anatomy. The deviations from the planned implant location to the final implant location for the dentate models were 0.89° ± 0.35° in angular deviation and 0.38 ± 0.21 mm in global apex position [31]. Furthermore, both studies by Block et al. (2017) and Nickenig et al. (2009) found that the X-Guide system outperforms freehand methods in implant placement accuracy [33,34]. The prospective study by Block et al., involving 478 patients and 714 implants, reported a mean angular deviation of 2.97 ± 2.09°, a mean global platform position deviation of 1.16 ± 0.59 mm, and a mean global apical position deviation of 1.29 ± 0.65 mm [33]. In the study by Nickenig et al., the X-Guide achieved an average shoulder deviation of 0.9 mm and an apex deviation of 0.6-0.9 mm, while the freehand method had deviations of 2.4-3.5 mm at the shoulder and 2.0-2.5 mm at the apex [34]. 

In the study by Wang et al. in 2022, using the X-Guide system, a total of 72 implants were placed by three experienced practitioners and three novice practitioners. The mean entry deviation of both experienced and novice practitioners was ~1.12 mm, the mean apex 3D deviation was ~1.66 mm, and the mean apex vertical deviation was ~0.57 mm. The angular deviation ranged from 1.25° to 6.68°. These minimal deviations highlight the X-Guide system’s superior precision. Although the study by Wang et al. focuses on how experience affects dental implant placement, the results showed no significant differences between experienced and novice practitioners [35].

Navigation-guided surgeries face several challenges, including human errors like minor hand tremors and perceptual inaccuracies, which can impact procedural precision. Device-related issues, like failures of infrared cameras, data cables, or monitor carts, pose significant obstacles. A complete breakdown of the navigation system will force the procedure being completed freehand. This underscores the importance of thorough preoperative checks to ensure system reliability [36]. 

Yomi dental robot

The Yomi dental robot is the first FDA-cleared haptic robotic-guided system for dental procedures in March 2017. The robotic system integrates haptic feedback with robotic guidance, providing tactile sensations to the surgeon to maintain precise control over surgical instruments. During surgery, Yomi offers real-time feedback on location, angulation, and depth, ensuring alignment with pre-planned implant positions. This system combines the physical constraints of a surgical guide with the real-time feedback of dynamic navigation systems [19,37].

**Figure 5:**
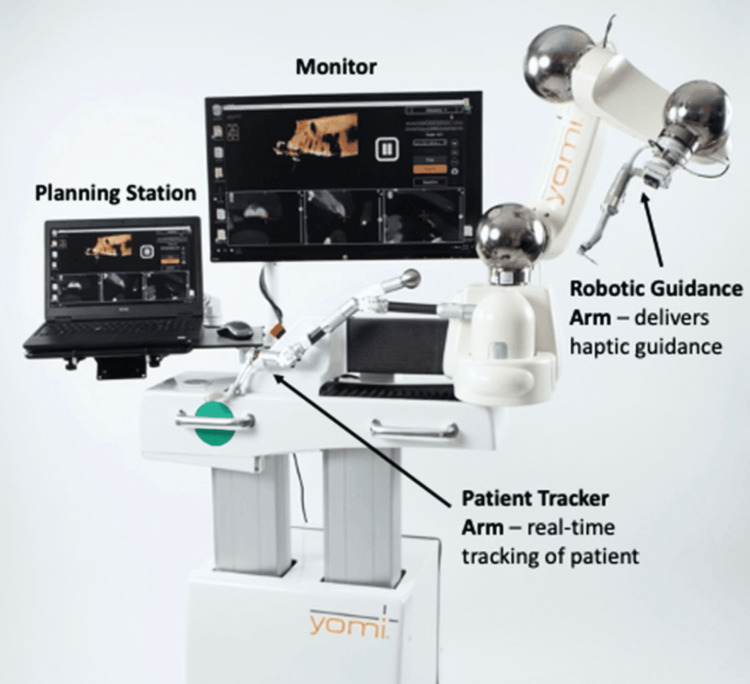
The Yomi system with an outline of its design and key features. Reproduced without modification from reference [38] under the Creative Commons License (CC BY 4.0). For further details, please visit the Creative Commons License (https://creativecommons.org/licenses/by/4.0/).

The Yomi system comprises several essential components, as can be seen in Figure 5. The planning laptop and monitor are utilized for pre-operative planning and continuous monitoring throughout the procedure. The tracker arm is meant for real-time anatomical mapping of the patient to a virtual model, enabling dynamic adjustments to patient movements and ensuring precise instrument alignment without requiring patient immobilization or continuous line-of-sight infrared tracking. For the Robotic Guidance arm, the surgeon maneuvers the robotic handpiece, which is attached to the robotic guidance arm, with high precision towards the pre-planned implant position accurately [19,37].

Using bite registration, the Yomi workflow begins by securing a surgical splint to the opposite side of the dental arch where the implant will be placed. Once a fiducial marker is attached, a CBCT scan is conducted to align the splint with the patient's dental anatomy. Implant planning is completed using Yomi’s software, which allows for precise mapping of implant location and critical structures. The software also aids in planning the final prosthetic restoration. During the procedure, Yomi’s robotic arm, attached to the splint, tracks the patient’s position. Once a pre-selected landmark is confirmed on the CBCT scan, the robot’s guidance arm and drill are locked into place based on the digital plan. Live visualization on the monitor ensures precise execution, while intraoral checks confirm the accuracy of each step in the procedure [39].

Yomi presents several advantages in dental implantology, sharing many benefits with the X-guide. Yomi provides real-time feedback during implant placement, ensuring precise control, and does not require a specific drill or implant. Additionally, surgery can be done on the same day, and Yomi has the capability for continuous use without rest, which reduces the risk of human error due to fatigue [19]. The system’s design improves ergonomics for the surgeon, with fewer obstructions such as surgical guides and drill keys. 

Yomi’s stability as a robotic system has demonstrated its ability to reduce inaccuracies in implant placement. In a clinical study conducted by Dr. Neugarten in 2023 involving 108 patients and 273 implants, the Yomi robot demonstrated high accuracy relative to the pre-operative plan with a mean angular deviation of 1.42° and minimal deviations of 1 mm. Specifically, the mean coronal and apical deviations were 1.10 mm and 1.12 mm, respectively, significantly lower than the freehand and static guided method. The discussion noted no significant differences in deviations between the mandible and maxilla [19]. Similarly, in a publication by Dr. Ali in 2023, Yomi was used to place six implants in the maxilla and five in the lower mandible in a 61-year-old female patient. All the implants were placed with a high degree of accuracy compared to the pre-operative plan, as reported by the mean angular deviation of 2.58°, the mean global coronal of 0.98 mm, and the mean global apical of 1.06 mm [39]. Furthermore, in a study by Bolding et al. published in 2021, 23 implants were placed in the mandible and 15 in the maxilla between five participants. The mean angular deviation was 2.56°, with a global coronal deviation of 1.04 ± 0.70 mm from the plan and a global apical deviation of 0.95 ± 0.73 mm. The signed depth deviation was 0.42 ± 0.46 mm proud [40].

Despite these benefits, Yomi does have some drawbacks. An experienced dentist must continuously monitor the robot, as it does not replace a surgeon's judgment, experience, or decision-making skills. Patient acceptance can vary significantly due to unease about robotic-assisted surgeries. The price of Yomi in the United States is approximately $200,000, making it inaccessible for many dental professionals [16]. Although additional long-term clinical studies are needed to evaluate outcomes and compare them with non-robotic methods, haptic robotic preparation offers significant intraoperative benefits for treating completely edentulous arches [40]. 

Differences between Yomi and X-Guide

When considering advanced dental implant systems, understanding the distinctions between Yomi and X-Guide is crucial for dentists to determine which technology aligns best with their practice needs and patient outcomes. The technology types are distinct. With Yomi, the robotic arm physically restricts the drill and offers resistance to incorrect movements, while also providing visualization on the monitor. In contrast, the X-Guide only displays real-time guidance on a monitor, leaving it up to the surgeon to manually maintain the correct trajectory. In terms of cost, both systems are notably expensive, with the X-Guide incurring high expenses primarily due to its advanced hardware components. However, the Yomi system is even more costly, often rendering it inaccessible for many dental practitioners. Additionally, both Yomi and X-Guide present a considerable learning curve for implant surgeons. 

When comparing the accuracy of both systems, it is clear that both Yomi and X-Guide provide significant improvements in precision over freehand techniques and static guides. Notably, the two largest sample size studies were Dr. Neugarten's study with Yomi, which showed a mean angular deviation of 1.42° across 273 implants, and the study by Block et al. with the X-Guide, which reported a mean angular deviation of 2.97 ± 2.09° for 714 implants [19,33]. The slightly higher deviation observed with the X-Guide may be influenced by its sample size, which was nearly 40% larger. Despite this, both systems maintained deviations around 1 mm, showcasing their high precision. These findings suggest that both Yomi and X-Guide are effective, reliable, and capable of delivering accurate results across a wide range of cases, making them suitable for clinical applications requiring precise implant placement. As technology continues to advance, enhancements in software and reductions in costs could make robotic systems, such as Yomi, more affordable, which may lead to their broader adoption in dental implantology.

Complex cases

Dental implant surgeries involving simultaneous sinus augmentation are challenging due to the anatomical complexities surrounding the maxillary sinus and the limited native bone available. These complex cases require precision to avoid perforating the sinus membrane [40]. 

One such case, detailed by Dr. Mergelmyer (2022), involved a 67-year-old male patient with less than 5 mm of native bone in the furcation of the tooth and concurrent left maxillary sinusitis. The tooth had been deemed unrestorable after an endodontic evaluation and was removed. Due to the limited bone height, an open (Caldwell-Luc) left maxillary sinus augmentation was performed to prepare for implant placement. A full-thickness flap was elevated under IV sedation. Using Yomi robotic guidance, a lateral window approach was created. The Schneiderian membrane was carefully elevated to accommodate the graft material. The dental implant osteotomy was completed using the Straumann protocol [39].

In another case by Dr. Mergelmeyer (2022), a 56-year-old male patient presented with a missing tooth #14, which was previously extracted due to a vertical fracture. Despite only 7 mm of bone height and a large mucus retention cyst in the left maxillary sinus, implant placement was planned using Yomi robotic guidance. A closed sinus lift (Summers' Technique) was performed with simultaneous placement of a 4.8 x 10 mm implant. The sinus floor was elevated by 3 mm, and graft materials were applied. Post-operative imaging showed successful bone augmentation [39].

Similarly, full-arch rehabilitation in patients with atrophic, edentulous jaws poses significant challenges due to the limited bone quality and quantity, making implant placement more complex. A 2020 study by Lopes et al. presented a case of a 57-year-old female who required maxillary full-arch rehabilitation to address both aesthetic and functional concerns. The All-on-4 treatment was utilized which involved placing two anterior axial implants and two posterior tilted implants. Using the X-Guide, real-time guidance helped avoid key anatomical structures like the maxillary sinus and nasal cavities. After tooth extraction and bone regularization, four NobelParallel CC implants were placed with insertion torques above 35 N/cm, allowing for the immediate connection of a provisional prosthesis. This approach achieved both immediate functionality and aesthetic improvement on the same day, showcasing the success of the dynamic navigation-assisted workflow [41].

**Figure 6:**
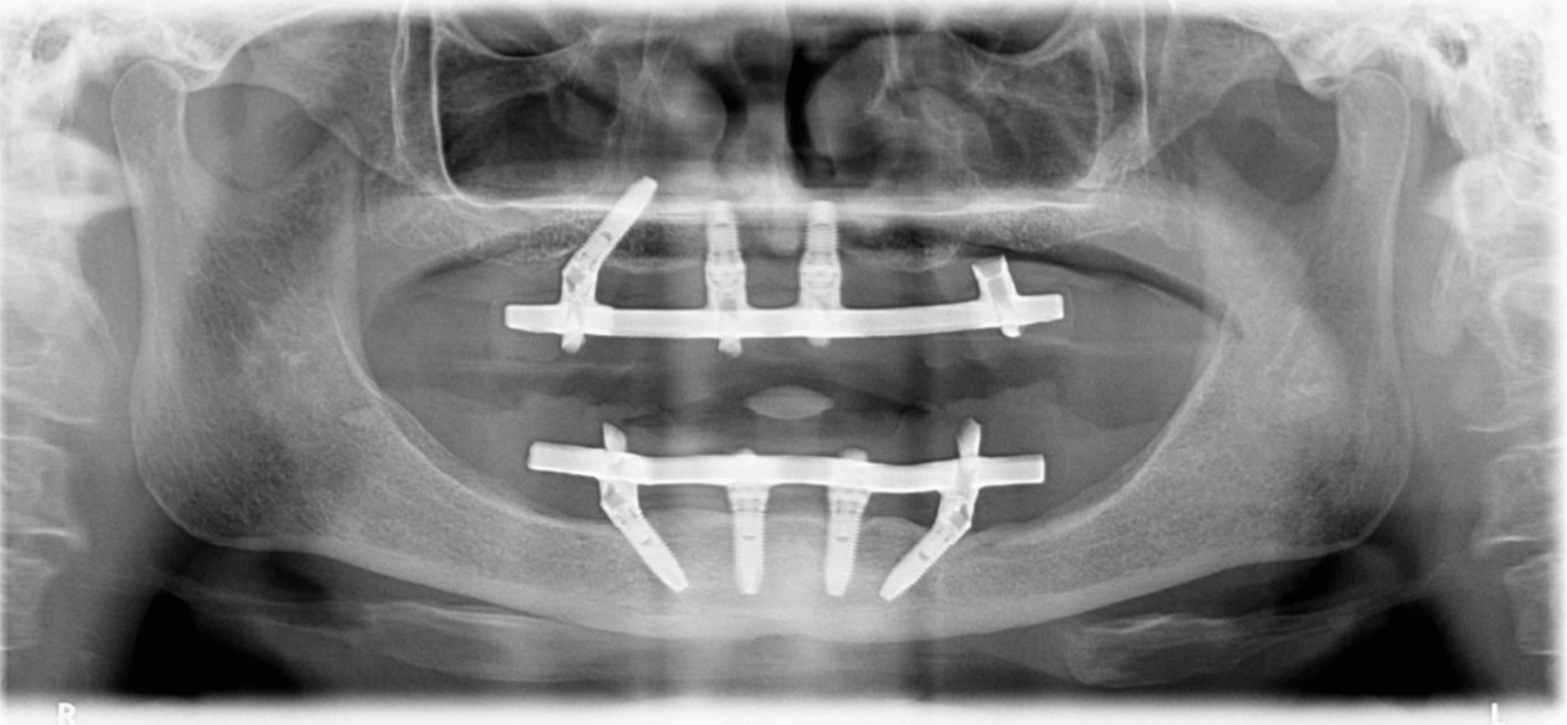
A preoperative orthopantomogram of the maxilla, indicating the absence of a posterior tilted implant in the third sextant. Reproduced without modification from reference [41] under the Creative Commons License (CC BY 4.0). For further details, please visit the Creative Commons License (http://creativecommons.org/licenses/by/4.0/).

In another challenging case report in the study by Lopes et al., a 70-year-old hypertensive female patient required the completion of her maxillary full-arch rehabilitation after the failure of a previously placed tilted implant, as can be seen in Figure 6. Due to the insufficient residual bone in the posterior maxilla, classified as D-V under the Cawood and Howell system, a zygomatic implant was chosen. She was using the All-on-4 Hybrid rehabilitation concept, which combined three standard implants with one zygomatic implant. The X-Guide dynamic navigation system was employed to ensure precise placement, avoiding critical structures like the infra-orbital nerve and base of the orbit. A NobelZygoma 45 mm implant was successfully inserted with an insertion torque over 50 N/cm. This approach provided a reliable and functional solution for full-arch rehabilitation, although opportunities for improvement in the fiducial markers protocol were noted to streamline the surgical process [41]. 

Endodontic procedures, like root-end resection, are highly complex due to their precision-dependent nature. This surgery's intricate nature demands removing all ramifications and lateral canals. Success rates fluctuate from 17% to 96%, mainly because the techniques require meticulous execution to ensure optimal outcomes [42]. A study by Martinho et al. explored whether three-dimensional dynamic navigation systems, specifically using X-Guide software by X-Nav Technologies, could enhance accuracy and efficiency in osteotomy and root-end resection. The study involved both novice and experienced endodontists and revealed that the X-Guide could significantly reduce accuracy deviations while halving procedure times. While novices couldn’t match the precision of experienced endodontists, the X-Guide System notably improved their performance, highlighting its efficacy in complex endodontic cases [43].

## Conclusions

Robotic implant surgery has increased the accuracy of implant placement and thus possibly lowering failure rates or the prevalence of peri-implant disease. Implants placed using a mucosa-supported drilling template demonstrated 3.5 times fewer inadequately positioned implants compared to the freehand method. Additionally, studies have shown that dental implant robots in China have shown excellent outcomes with low mean entry deviations, and this can also be seen in X-Guide and Yomi achieving small deviations from the pre-planned positions.

While these advancements already show significant promise in areas such as endodontics, ongoing research and development are essential to fully realize the potential of robotic systems in broader, specialized fields of dentistry. Expanding their applications could profoundly impact patient outcomes and pave the way for further innovation in dental care.
